# Focus on foot and ankle

**DOI:** 10.1007/s00068-021-01726-9

**Published:** 2021-07-29

**Authors:** Thomas Mittlmeier

**Affiliations:** Department of Trauma, Hand and Reconstructive Surgery, Rostock University Medical Center, Rostock, Germany

Dear Reader,

Ankle fractures represent common injuries. Correspondingly, the treatment of ankle fractures is routine in hospitals of any level of care. For decades standard radiographic techniques were employed as the sole diagnostic tool and traditional nonoperative and surgical strategies according to AO/OTA recommendations at that time were believed to suffice for a generally favourable outcome [1]. An even cursory view at the contemporary literature reveals on the one side that there is a lack of studies with high evidence despite the high incidence of ankle fractures and on the other side that the long-term outcome in ankle fractures is at best moderate [2, 3]. Involvement of the syndesmotic and/or the delta ligament complex and bi-, tri- and quadrimalleolar fracture types have a remarkably poor prognosis, in particular, if anatomic reduction of any fracture component and restoration of ligamentous stability are missed [4]. With the advent and consecutive spread of spatial analysis employing CT scanning the detection of hidden fracture components, the understanding of the injury pattern of the ankle mortise and the relevance of anatomic reduction have considerably increased [5]. Consequently, this lead to the postulate of a postoperative CT control as an appropriate measure to confirm the quality of reduction in complex fracture patterns. Further, the spatial analysis promoted a better comprehension of the biomechanical dimensions of distinct fracture components, e.g., the posterior malleolus, encompassing a paradigm shift and modified surgical techniques [4].

The intraoperative application of cone beam CT in cases with a complex fracture pattern and unstable mortise allowed for substituting the postoperative CT by intraoperative assessment of adequate reduction and eventual correction of non-anatomic reduction during the same procedure rendering a second-step surgical revision unnecessary [6].

Until now, fibular rotation and maltorsion of the fibula after ankle fracture with concomitant lesion of the syndesmotic complex has gained limited attendance in the literature, only. Adequate determination of fibular rotation is one of three topics of research provided by Sven Vetter et al. [7]. Their group from Ludwigshafen/Germany was one of the first worldwide to study intraoperative 3-D geometry of the foot and ankle joint via cone beam CT [6]. In the first manuscript of our focus edition they studied 100 healthy ankle joints to define the most appropriate location to measure fibular rotation. They now recommend to assess fibular rotation at a level of 6 mm below the ankle joint line with the utmost reliability and reproducibility compared with neighboring alternative locations for measurement [7].

Their second paper [8] addresses the intraoperative use of cone beam CT to evaluate anatomic reduction in ankle fractures with unstable syndesmotic injuries. At a mean follow-up of 6 years they found inferior outcomes and a higher rate of posttraumatic osteoarthritis in patients with incomplete syndesmotic reduction compared with those with anatomic reduction. In the third publication from the Ludwigshafen group a cadaver study had been performed employing 22 cadaver legs where Vetter et al. [9] dissected the anterior part of the syndesmotic complex, the interosseous membrane and, additionally, made an osteotomy of the posterior malleolus. They found that in the unloaded situation corresponding to the intraoperative condition neither the release of the syndesmosis nor the osteotomy of the posterior malleolus led to a manifest fibular malposition detectable during cone beam CT. Therefore, they hint at potentially unstable syndesmotic lesions despite non-pathologic morphology in cone beam CT examinations which emphasizes the additional need for intraoperative provocation tests as the application of a rotational test or the Cotton/hook test.

Michal Tuček and colleagues examined the clinical outcome following surgical treatment of ankle fractures with a large posterior malleolar fragment corresponding to a Bartoníček/Rammelt type 4 fracture [10]. A CT scan was available before and after surgical reconstruction demonstrating anatomic reconstruction in nine patients, only. Less than 3 years after surgery the patients with an anatomic reduction of the posterior malleolus exhibited the best functional results assessed via the AOFAS hindfoot score. Two out of six patients with posttraumatic osteoarthritis revealed either a malrotation of the fibula, a step-off at the joint line after reduction of the posterior malleolus or a widening of the syndesmosis.

Finally, Bi and colleagues examined the diagnostic value of the intraoperative tap test for acute deltoid ligament instability which had been proposed by Rajagopalan et al. [12]. In a patient collective with 92 ankle fractures they compared the tap test versus the gravity stress test and performed an open dissection of the deltoid ligament [11]. In particular, the negative predictive value of the tap test with 100% is superior to the gravity stress test with 95.6%.

In summary, the manuscripts from our current focus topic underline the notion that the treatment of ankle fracture is rapidly further developing towards a more predictable outcome. I do hope that the lecture facilitates the integration of these findings into your daily work.
